# Inadvertent Ingestion of Orthodontic Wire Impacted in the Esophagus, Mistaken for a Chicken Bone

**DOI:** 10.7759/cureus.75396

**Published:** 2024-12-09

**Authors:** Alasdair W Mayer, Assem Shayah

**Affiliations:** 1 ENT, York Teaching Hospital, York, GBR

**Keywords:** accidental, esophageal foreign body, orthodontic appliance, orthodontic arch wire, rigid esophagoscopy

## Abstract

Accidental ingestion of foreign bodies frequently necessitates emergency department visits, with many cases requiring surgical consultation. Although most ingested items pass through the gastrointestinal tract uneventfully, orthodontic components, such as wires, present a specific risk due to their shape and material properties. This report describes a rare case of a 13-year-old male adolescent whose initial presentation suggested ingestion of a chicken bone. He was later found to have an orthodontic archwire impacted in the esophagus, which was successfully removed via rigid esophagoscopy. This case highlights the need to consider orthodontic devices as potential foreign bodies in adolescents, emphasizing that such objects may be ingested inadvertently. Prompt imaging and intervention are critical to prevent complications, such as obstruction or perforation, ensuring patient safety and optimal clinical outcomes.

## Introduction

Accidental ingestion of foreign bodies frequently necessitates emergency department visits and subsequent surgical consultations. Although most ingested foreign bodies pass through the gastrointestinal tract without issue, approximately 10%-20% of cases require endoscopic intervention, and 1% require surgical removal [1]. The ingestion of dental foreign bodies is most commonly observed in the elderly, primarily due to the widespread use of dentures in this demographic [2,3]. In contrast, among younger patients, ingestion typically involves components of orthodontic appliances; however, cases requiring urgent intervention are rarely reported [4]. To our knowledge, this paper presents the first documented case of an adolescent inadvertently ingesting an orthodontic component that required emergency management.

## Case presentation

**Figure 1:**
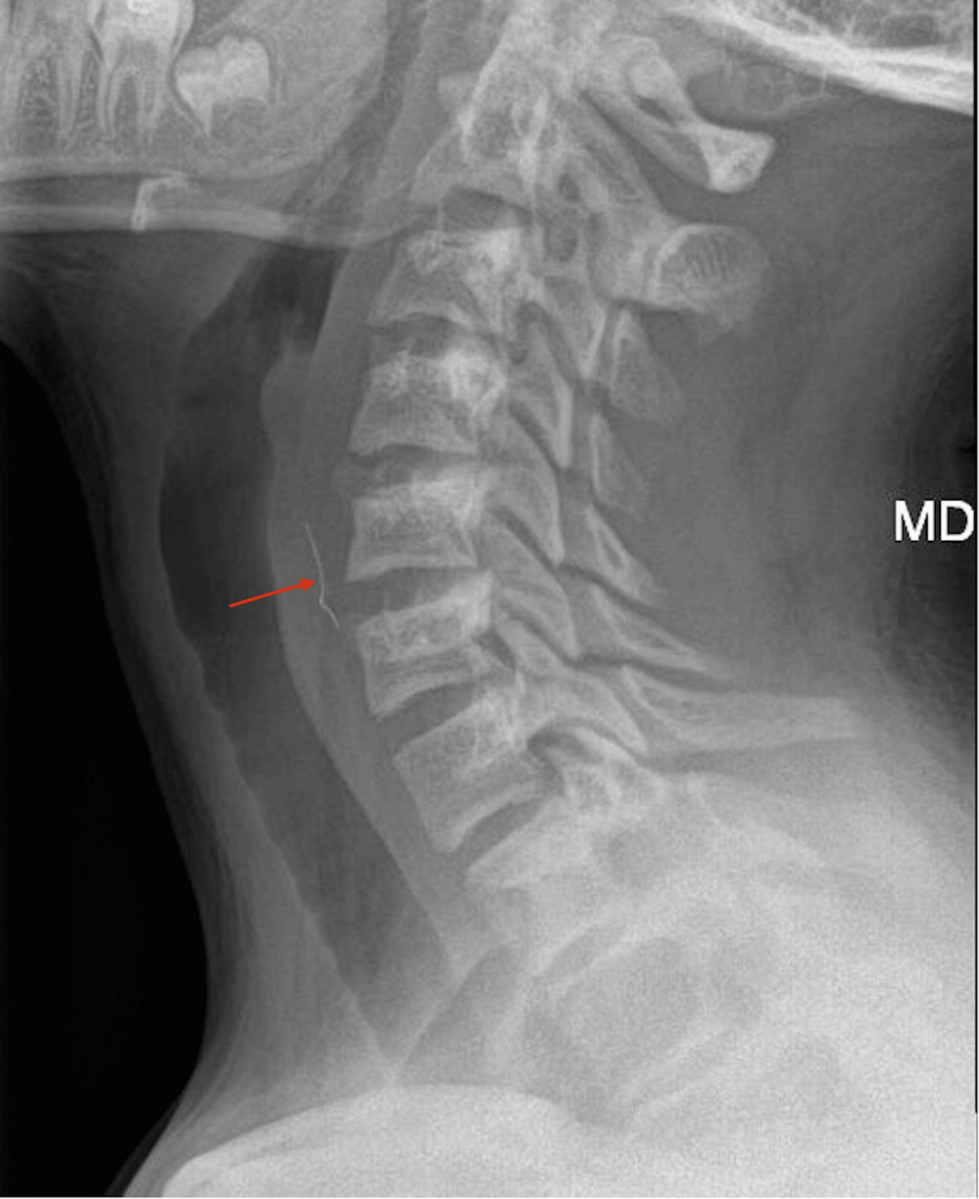
Lateral soft tissue neck X-ray demonstrating a linear opacity projected over the esophagus at the C5-C6 vertebrae level (red arrow).

A 13-year-old boy presented to the emergency department of a district general hospital with a complaint of a chicken bone lodged in his throat. The incident occurred earlier that evening while eating home-cooked chicken with bones, during which he experienced sharp pain in his throat. Subsequently, he was unable to swallow solids but could manage small sips of liquids. He reported moderate odynophagia but was otherwise systemically well, with normal vital signs. He had no chest pain, back pain, fever, hemoptysis, or hematemesis. His medical history was unremarkable, and he was normally fit and well. Physical examination revealed no abnormalities, including the absence of surgical emphysema. A lateral soft tissue neck radiograph showed a linear opacity, approximately 17 mm in length, overlying the esophagus at the C5-C6 vertebrae level (Figure 1).

**Figure 2:**
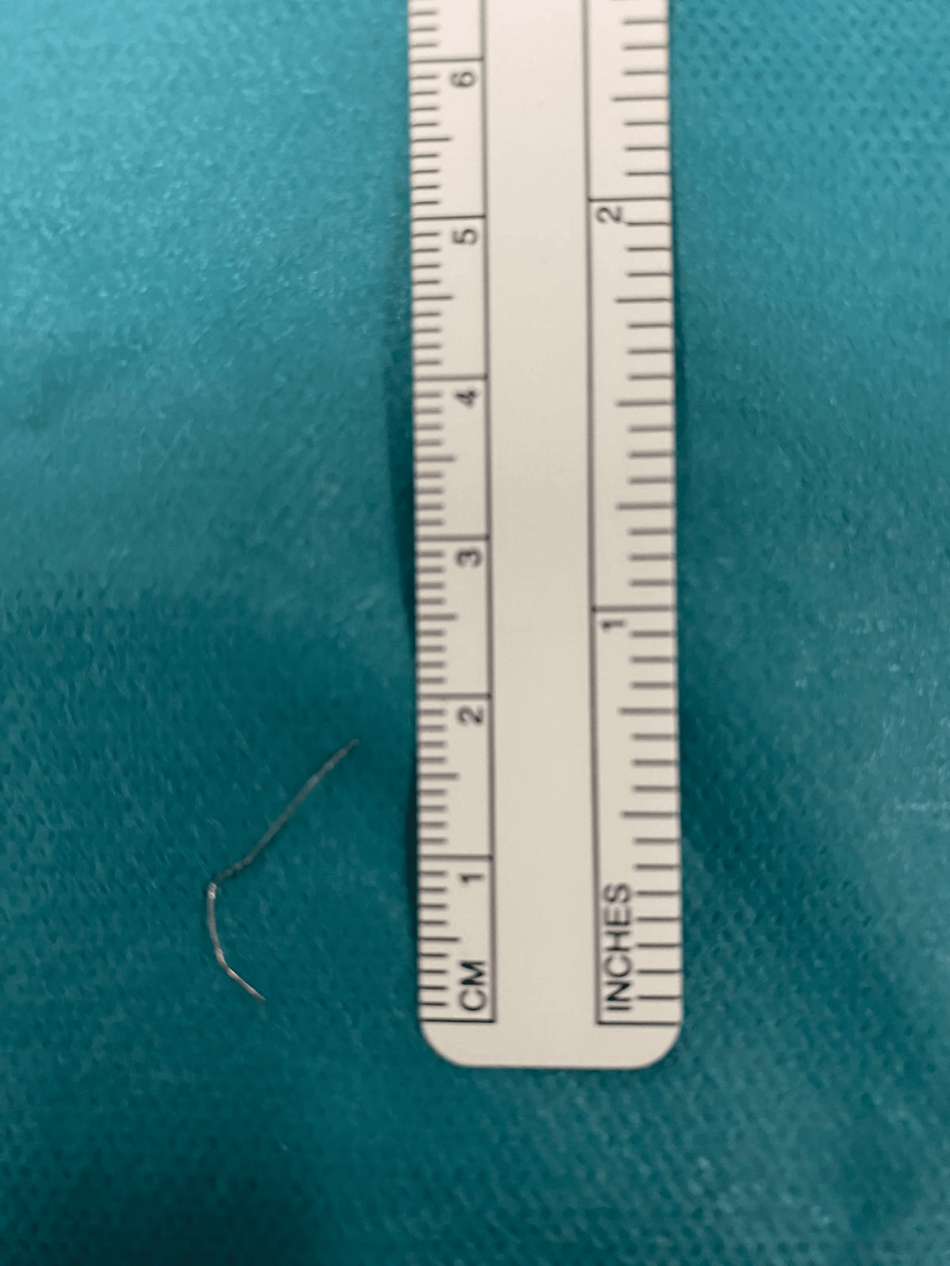
Segment of orthodontic archwire that was removed from the patient's cervical esophagus.

The patient was transferred for an urgent ENT assessment and evaluated approximately four hours after the onset of symptoms. He reported a gradual increase in the severity of pain with swallowing, but no other changes in his condition. Due to the severity of his symptoms and the radiographic findings, he was taken to the operating room for emergent rigid esophagoscopy under general anesthesia. During the procedure, a piece of metal wire was found impacted in the cervical esophagus, 17 cm from the incisors. The wire was successfully removed with forceps (Figure 2).

Subsequent examination of the esophagus revealed minor mucosal lacerations at the impaction site, with no evidence of perforation. Notably, the wire matched a missing segment of archwire from the patient’s dental braces. Further discussion with the patient’s mother revealed that he had visited his orthodontist for a brace adjustment two weeks earlier. Postoperatively, the patient was kept nil by mouth for eight hours and was discharged the following morning after resuming oral intake without complications.

## Discussion

In cases of accidental ingestion of dental foreign bodies, more than 80% are located in the upper gastrointestinal tract [3]. Among various orthodontic appliances, orthodontic wires are frequently the ingested component [4]. These ingestions can occur during wire placement when the distal end is trimmed to protect the oral mucosa or during everyday activities, particularly when subjected to masticatory forces [4]. Despite the potential severity, cases requiring emergent management are rarely reported in the literature [4]. Most documented cases involve adolescent patients [5-8] and commonly occur during eating [5,6,9,10]. While previous reports have noted orthodontic wire ingestion as the presenting complaint, in our case, it was mistakenly identified as food by the patient, highlighting the risk of inadvertent ingestion. Greater awareness of this possibility could have prompted a more thorough medical history, including recent dental procedures, and an examination of the patient’s orthodontic appliance for any missing components. Such measures may have facilitated earlier identification of the foreign body and prevented diagnostic confusion.

While some cases have been managed by monitoring with serial radiographs, allowing the wire to pass spontaneously without complications [6,7], others have required endoscopic intervention for removal from locations such as the hypopharynx [9], esophagus, gastric antrum [10], and pylorus [5]. Additionally, one reported case required emergency laparotomy due to a small bowel perforation caused by a 20-mm piece of archwire [8].

In 2022, the British Orthodontic Society issued guidelines for managing ingested foreign bodies [11]. These guidelines indicate that most ingested orthodontic objects (80%-90%) pass through the digestive tract spontaneously without complications within four to six days, as they are typically small and smooth. However, objects that are sharp, longer than 5 cm, or wider than 2 cm may fail to pass through the pyloric sphincter or cause obstruction and potential perforation in areas such as the duodenum, ileocecal valve, or sigmoid colon. Initial investigations typically include chest and abdominal radiographs, with a lateral neck radiograph recommended if a foreign body is detected in the neck on a chest radiograph. A computed tomography scan may be warranted if the object’s location is unclear or if perforation is suspected based on clinical or radiological evidence. Management depends on whether the patient is symptomatic. Symptomatic patients, particularly those with a potentially hazardous object in the upper gastrointestinal tract or duodenum, should undergo gastrointestinal endoscopy as the primary treatment. If the object has moved past the duodenum, clinical observation, administration of a laxative, and repeated radiography may be necessary. Surgical intervention is rare, with gastrointestinal perforation occurring in less than 1% of cases. For asymptomatic patients who are tolerating oral intake well and whose ingested object is not considered dangerous, discharge with instructions to return to the emergency department for symptoms like fever, abdominal pain, vomiting, or blood in the stool is appropriate. If the ingested object is deemed hazardous, outpatient follow-up should be arranged, even if the patient is asymptomatic.

## Conclusions

This report highlights that orthodontic components can be inadvertently ingested, emphasizing the need for clinicians to consider dental or orthodontic appliances when assessing suspected foreign body ingestion. Incorporating this consideration into routine history-taking and examination is crucial, especially given the risks posed by certain materials. While such awareness is common in elderly patients with dentures, it can be easily overlooked in children and adolescents. The successful resolution of this case, with the removal of the wire without any subsequent complications, demonstrates the effectiveness of adhering to the latest guidelines issued by the British Orthodontic Society. A tailored management strategy, based on the patient's symptoms and the nature of the ingested object, may range from conservative monitoring to urgent endoscopic or surgical removal. Rapid diagnostic imaging and timely clinical interventions are essential to prevent serious complications, such as obstruction or perforation, thus ensuring patient safety and optimal outcomes.
